# Public Attitudes During the Second Lockdown: Sentiment and Topic Analyses Using Tweets From Ontario, Canada

**DOI:** 10.3389/ijph.2022.1604658

**Published:** 2022-02-21

**Authors:** Shu-Feng Tsao, Alexander MacLean, Helen Chen, Lianghua Li, Yang Yang, Zahid Ahmad Butt

**Affiliations:** ^1^ School of Public Health Sciences University of Waterloo, Waterloo, ON, Canada; ^2^ Systems Design Engineering, University of Waterloo, Waterloo, ON, Canada; ^3^ Faculty of Science, University of Waterloo, Waterloo, ON, Canada

**Keywords:** social media, COVID-19, sentiment analysis, lockdown, topic modelling, mask, vaccine

## Abstract

**Objective:** This study aimed to explore topics and sentiments using tweets from Ontario, Canada, during the second wave of the COVID-19 pandemic.

**Methods:** Tweets were collected from December 5, 2020, to March 6, 2021, excluding non-individual accounts. Dates of vaccine-related events and policy changes were collected from public health units in Ontario. The daily number of COVID-19 cases was retrieved from the Ontario provincial government’s public health database. Latent Dirichlet Allocation was used for unsupervised topic modelling. VADER was used to calculate daily and average sentiment compound scores for topics identified.

**Results:** Vaccine, pandemic, business, lockdown, mask, and Ontario were six topics identified from the unsupervised topic modelling. The average sentiment compound score for each topic appeared to be slightly positive, yet the daily sentiment compound scores varied greatly between positive and negative emotions for each topic.

**Conclusion:** Our study results have shown a slightly positive sentiment on average during the second wave of the COVID-19 pandemic in Ontario, along with six topics. Our research has also demonstrated a social listening approach to identify what the public sentiments and opinions are in a timely manner.

## Introduction

The Coronavirus disease (COVID-19) pandemic has persisted for more than a year and resulted in over 141 million infections with over 3 million deaths worldwide [1]. In Canada, it has led to over 1 million positive cases and caused more than 24,000 deaths [2]. During this global crisis, when people have been forced to stay at home and connect virtually, social media platforms have played an increasingly significant role in communications now more than ever before. Therefore, social media data, such as tweets, have become even more important in health research associated with the current pandemic. Understanding public discourses and sentiments from social media data has been critical for researchers and decision makers since it correlates with our behaviours that help or fail to eliminate the COVID-19 infections. Scholars have conducted various topic modelling and sentiment analysis to understand public concerns or attitudes toward the pandemic and public health measures, such as mask wearing, handwashing, travel restrictions, and lockdowns since the early pandemic [3–6]. For example, Abd-Alrazaq et al identified 10 themes with positive sentiments and two topics with negative sentiments from 2.8 million English tweets between February 2 and March 15, 2020 [3]. Furthermore, Boon-Itt and Skunkan found topics changed over time, but negative sentiments persisted when analyzing almost 11 million English tweets from December 13, 2019, to March 9, 2020 [4]. Similarly, Chandrasekaran et al. collected 13.9 million English tweets posted by individuals between January 1 and May 9, 2020. Their findings show a consistent negative sentiment towards topics related to the spread and growth of COVID-19, origin of virus, political perspectives, and racial discrimination, whereas sentiments toward topics associated with preventive measures and treatments, economic impacts, government implementations, healthcare industry changed from negative to positive [5]. Additionally, Valdez et al. collected 86 million English tweets from the United States (US) between January 22 and April 9, 2020 and found the number of tweets and ranking of eight identified topics—“China, Trump, US, lockdown, pandemic, social distancing, home, deaths”—rose and fell over time with the overall sentiment steadily shifting from negative to positive [6].

Machine learning (ML) techniques have been applied to such research in topic modelling and sentiment analysis. For instance, Latent Dirichlet Allocation (LDA) is commonly applied to topic modelling, and Valence Aware Dictionary and sEntiment Reasoner (VADER) is widely used for sentiment analysis [4–6]. LDA identifies topics from documents by classifying relevant individual words or phrases together modelled by Dirichlet distributions [7].VADER is a lexicon and rule-based sentiment analysis tool that classifies words as positive or negative [8]. Such advanced ML approaches have gained popularity among quantitative studies in which scholars analyze a large volume of social media data. For example, the World Health Organization (WHO) has developed an Early AI-supported Response with Social Listening (EARS) to understand the public discourses in different countries with data from social media and Internet search queries using a semi-supervised ML algorithm [9].

Social media and Internet query data have also been used to predict the number of COVID-19 cases [10–13], especially in China, where about 1 billion of its 1.4 billion population have access to the Internet and social media [14]. Although methods to construct predictive models vary across these studies, researchers, in general, can correctly predict the number of COVID-19 cases in China 6–14 days before these cases are officially confirmed by lab tests [9–11]. On the other hand, Shen et al. found a ratio of 1:4 between the number of cases and social posts in Wuhan, China [13]. Additionally, social media data with self-disclosed geolocations have been used to explore the public adherence to social distancing to eliminate the COVID transmissions [15, 16] or the spread of the pandemic [17, 18].

Although recent literature has demonstrated the utility of social media data for various research, unsupervised ML approaches have not been studied as extensively as supervised ML techniques. In other words, data collection in current literature is conducted with pre-identified keywords or hashtags of research interest, and these pre-defined keywords have also been used as criteria for topic classifications [3–6, 10–18]. In addition, each country has its unique context that may not be reflected in global studies as scholars have collected and analyzed social media data limiting to English but not specific countries. In Canada, the second wave of COVID 19 pandemic occurred from December 5, 2020, to March 6, 2021, and Ontario implemented its second lockdown between December 26, 2020, and January 2021. Mask-wearing was mandated after the province moved out of the lockdown. However, in the United States (US), lockdowns were rarely implemented throughout the pandemic, except New York and California states. Futtheremore, mask-wearing wasn’t mandated. Jang et al. compared tweets from Canada with those from US and found that while Canadians shared some similar topics with Americans in factors associated with COVID-19 transmissions, US president, and Wuhan initial outbreak, Canadians expressed appreciation and border restrictions for travel, whereas Americans discussed the similarity between COVID-19 and influenza and lockdown impacts [19]. In the case of face mask-wearing, people in Asian countries have higher adherence to wearing masks as recommended by public health authorities than those in western culture [20].

While LDA and VADER have been commonly used for topic modelling and sentiment analysis, respectively, deep learning techniques, such as Long Short-Term Memory (LSTM) and Bidirectional Encoder Representations from Transformers (BERT), have become more successful and have been adopted in more recent literature for sentiment analysis [21–24]. For example, Chandra and Krishna [21] used deep learning models for COVID-19 tweet sentiment analysis in India from March to September 2021. The study has classified more granular 11 emotions than three general sentiments (i.e., positive, negative, and neutral) from the conventional VADER [21]. Similarly, Imran et al. [22] used a supervised multi-layer LSTM to classify and compare six emotions across Pakistan, India, Norway, Sweden, Canada, and the US [22]. Das and Kolya [23] also proposed a supervised deep convolutional neural network (CNN) to evaluate sentiments and predict COVID-19 cases globally since data were retrieved from 15 countries [23]. Yet they classified emotions expressed in tweets only as either positive or negative [23]. However, deep learning techniques still have some limitations. For instance, supervised deep learning techniques demonstrated in existing literature have been conducted in a supervised manner, and this requires large and correctly labelled training datasets [22–24]. Interpretability is another issue because public health professionals may not have deep understanding of deep learning models, which have been more complicated than conventional LDA and VADER. In other words, although deep learning has more optimal performance than conventional techniques, it has been a “black box” viewed by many public health professionals [24]. Additionally, compared to conventional methods, deep learning techniques generally require more computing power, leading to limited implementation in practice.

On the other hand, most studies conducted in the first wave analyzed data with a relatively shorter time frame, several weeks or 1 month, for example [3–6]. Furthermore, given existing literature, it still lacks follow-up research to investigate how sentiments and topics have changed over time after the first wave of the COVID-19 pandemic in Canada. Furthermore, given Canada’s cultural diversity and the greater provincial autonomy than the federal, each province has tackled the pandemic differently in Canada. It is of our research interest to investigate at a more focused, local level. Therefore, this study aimed to apply an unsupervised ML approach with minimal manual validations to explore both topics and corresponding sentiments using tweets from Ontario, Canada, from December 5, 2020, to March 6, 2021.

## Methods

### Tweet Collection

English tweets originated in Ontario, Canada, were collected between December 5, 2020, and March 6, 2021, without being filtered by COVID-19 related keywords or hashtags. This is because our study aimed to apply an unsupervised ML approach to identify possible patterns beyond those explicitly mentioning COVID-19 related keywords or hashtags. Instead, major metropolitan areas in Ontario, namely geolocation tags from Toronto and Ottawa, were identified to limit the scope of data collection to a level allowed by available resources. With these query parameters limited to two cities and the timeframe, 569,467 tweets from Toronto and 141,469 tweets from Ottawa were returned from December 5, 2020, to March 6, 2021, *via* the Twitter Developer API [25].

### Public Health Policy Data Collection

Data were collected on Ontario public health policy changes by each public health unit as a means of comparison against daily case changes and Twitter sentiment from December 5, 2020, to March 6, 2021. Public health policy changes include major vaccine approvals, lockdown announcements, school closures and other public health related enforcement of policies regarding COVID-19 by reviewing each public health unit’s COVID-19 information page within the applicable regions.

### Daily Case Count Collection

The daily case counts of all public health units in Ontario from December 5, 2020, to March 6, 2021 were retrieved from the Ontario provincial government’s public health database. The extracted data were organized by active cases, resolved cases, and deaths as defined by the Government of Ontario’s health unit.

### Data Processing

**FIGURE 1:**
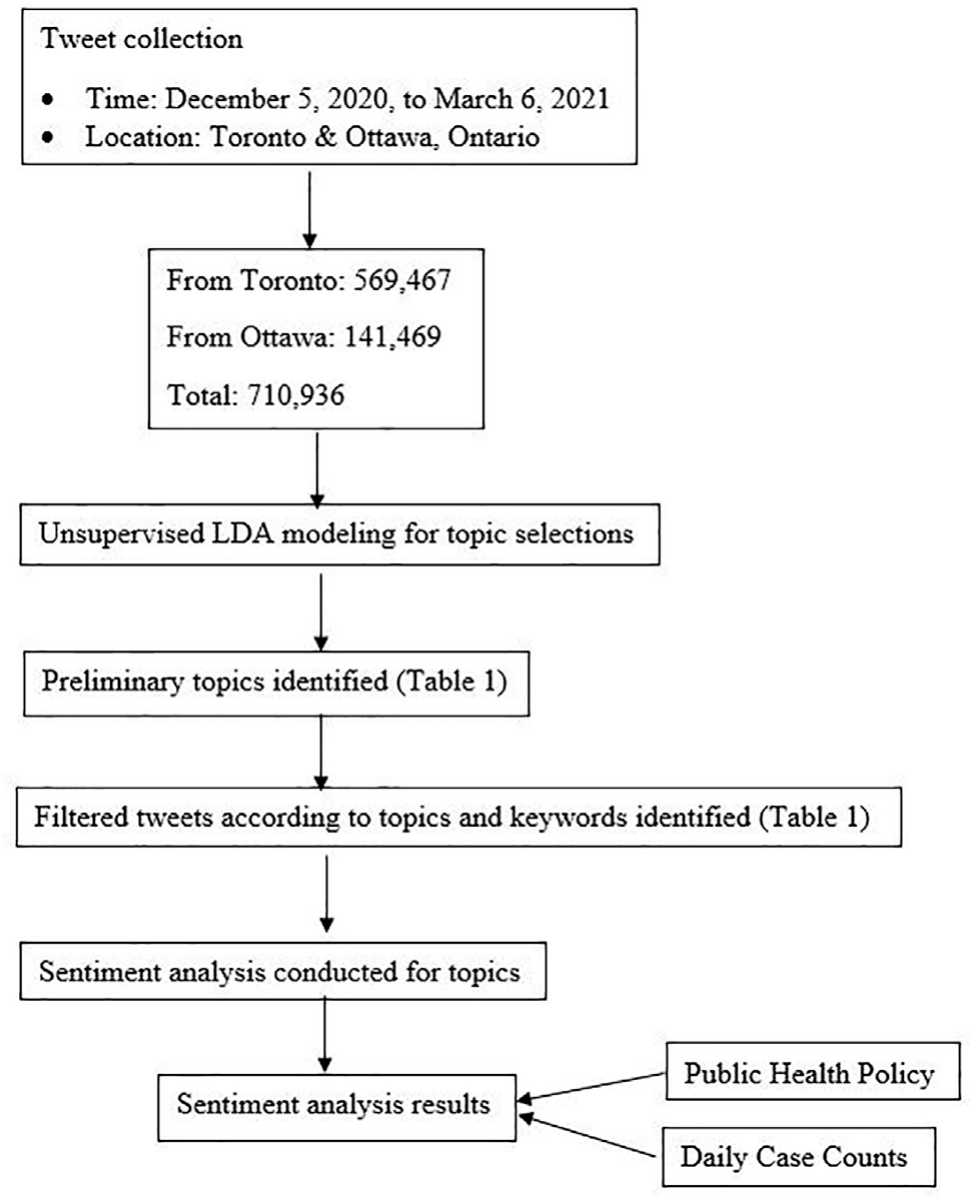
Flow diagram [Public Attitudes during the Second Lockdown: Sentiment and Topic Analyses using Tweets from Ontario, Canada, Canada, 2020].

Figure 1 shows the overall flow diagram. All tweets were transformed to lowercase. Next, non-texts were removed, including punctuations (“[”, “]”, “,”, “\”, “.”, “:”, “!”, “/”), special characters (“#”, “%”, “$”, “@”), uniform resource locator (URL), emoji, and stop words following standard data preprocessing procedure as previous studies [3–6]. Unsupervised LDA topic modelling was firstly applied by using Python’s Gensim package to generate potential keywords for each identified topic in previous literature [3–6]. Three researchers—S-FT, HC, and ZB—then reviewed the preliminary keywords for each topic generated by the unsupervised topic modelling to collaboratively interpret topics for further data cleansing before conducting the sentiment analysis. All disagreements were resolved through discussions among the three researchers. To reduce noise irrelevant to the pandemic, such as the 2020 United States presidential election, from the sample and to better understand the sentiment around various components of the public discourses in Ontario, Canada, the full dataset was further filtered down, and a subset was created by using the topics and keywords identified. The filtering was done by searching for substrings in a tweet matching one of the keywords for a given topic. In addition, to ensure that the sentiment score reflects public sentiment as closely as possible, emojis were added back to the tweets since VADER can recognise them when calculating sentiment scores [8]. Furthurmore, tweets from public health, governments agencies and political organizations were excluded in the subset as described in a previous study [26].

VADER is used to identify sentiment as it calculates the sentiment attached to individual tokens in text and is adjusted to understand specific tokens commonly used in English social media text [8]. For each tweet, VADER provides an empirically found sentiment value for individual terms, emoticons, and punctuation marks, modified by defined rules for combinations of terms. From these values, a compound overall sentiment score is calculated by taking the sum of the individual sentiments, with positive values for positive sentiments and negative values for negative ones. This raw score is normalized using the equation $Compound=\;\frac{RawScore}{\sqrt{RawScore^{2}+\alpha }}$ , where α is set to the value of 15 based on the maximum values seen by the authors in training sets and then normalized to ensure that the normalized value is indeed between −1 and +1 [8]. The compound score was therefore used for our sentiment analysis. However, it is difficult for humans to understand numeric sentiment scores. Therefore, the compound sentiment scores are categorized as “positive” if they are equal or greater than +0.05, “negative” if they are equal to or lower than −0.05 and “neutral” if neither based on prior research [27].

### Manual Validation

To determine the accuracy of the unsupervised LDA topic modelling and sentiment analysis. Three researchers—S-FT, HC, and ZB—conducted inter-rater manual validations for 3% of random tweets from each topic and their sentiment. S-FT was the primary rater, HC and ZB served as secondary raters. The 3% random sample is calculated according to Krippendorff’s sampling method with 10% probability of the rarest relevant instances and 95% desired significance level of the answers to our research question [28]. Inter-rater agreement percentage and validation results were reported in Supplement A.

## Results

Table 1 shows the topics given by the unsupervised LDA topic modelling. These topics were chosen based on the research questions, i.e. public sentiment with regard to lockdown policies and researchers’ interpretations with corresponding keywords and synonyms from each topic’s keyword outputs generated by the unsupervised LDA topic modelling and manual validation.

**TABLE 1:** Topics, keywords, and synonyms generated from the unsupervised LDA [Public Attitudes during the Second Lockdown: Sentiment and Topic Analyses using Tweets from Ontario, Canada, Canada, 2020].

Topics	Human interpretations	Keywords and synonyms	Number of tweets included
Vaccine	Opinions toward COVID-19 vaccine approval, access, availability, etc.	vaccine, vaccinations, vaccination, vaccines, vaccinated, immunization	6,932
Pandemic	Impacts of the COVID-19 on life	pandemic, covid-19, covid19, covid 19, covid_19, covid, sars, coronavirus, corona virus, corona, sars-cov-2, outbreak, cases	17,285
Business	Reviews, supports, and impacts of the COVID-19 pandemic on busniesses, markets, and economics	business, binesses, biness, businesses, biz	5,148
Lockdown	Opinions toward the second lockdown	lockdown, lockdowns, lock down, shutdown, shut down, shutdowns, grey zone	4,884
Mask	Opinions toward wearing masks	mask, wearing, wear, face cover, facial cover, facemasking, face mask, masks, maskwearing	6,893
Ontario	Things happended in Ontario	fordnation, ford, ontario	25,401

**FIGURE 2:**
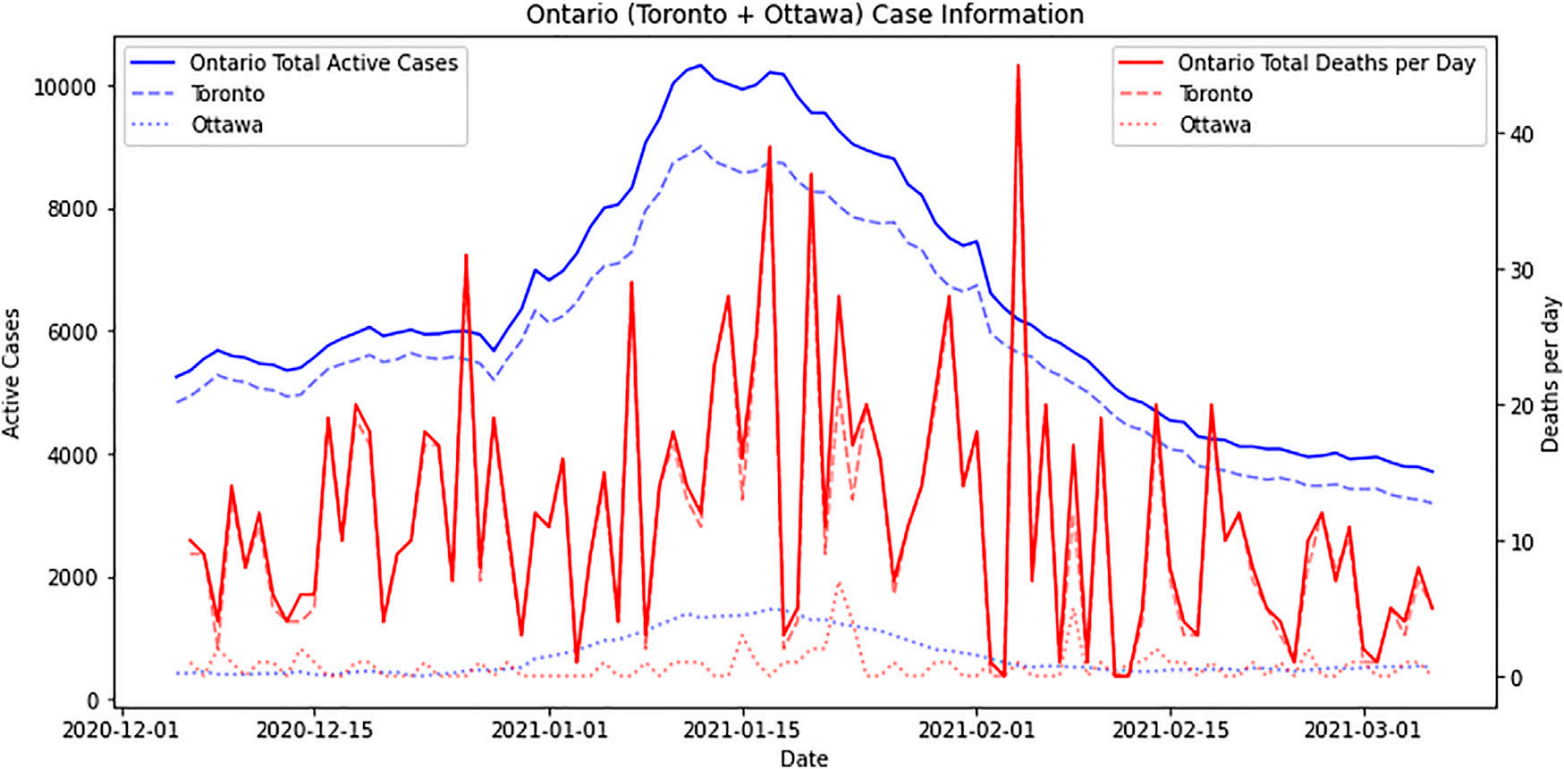
COVID-19 cases in Toronto and Ottawa [Public Attitudes during the Second Lockdown: Sentiment and Topic Analyses using Tweets from Ontario, Canada, Canada, 2020].

**FIGURE 3:**
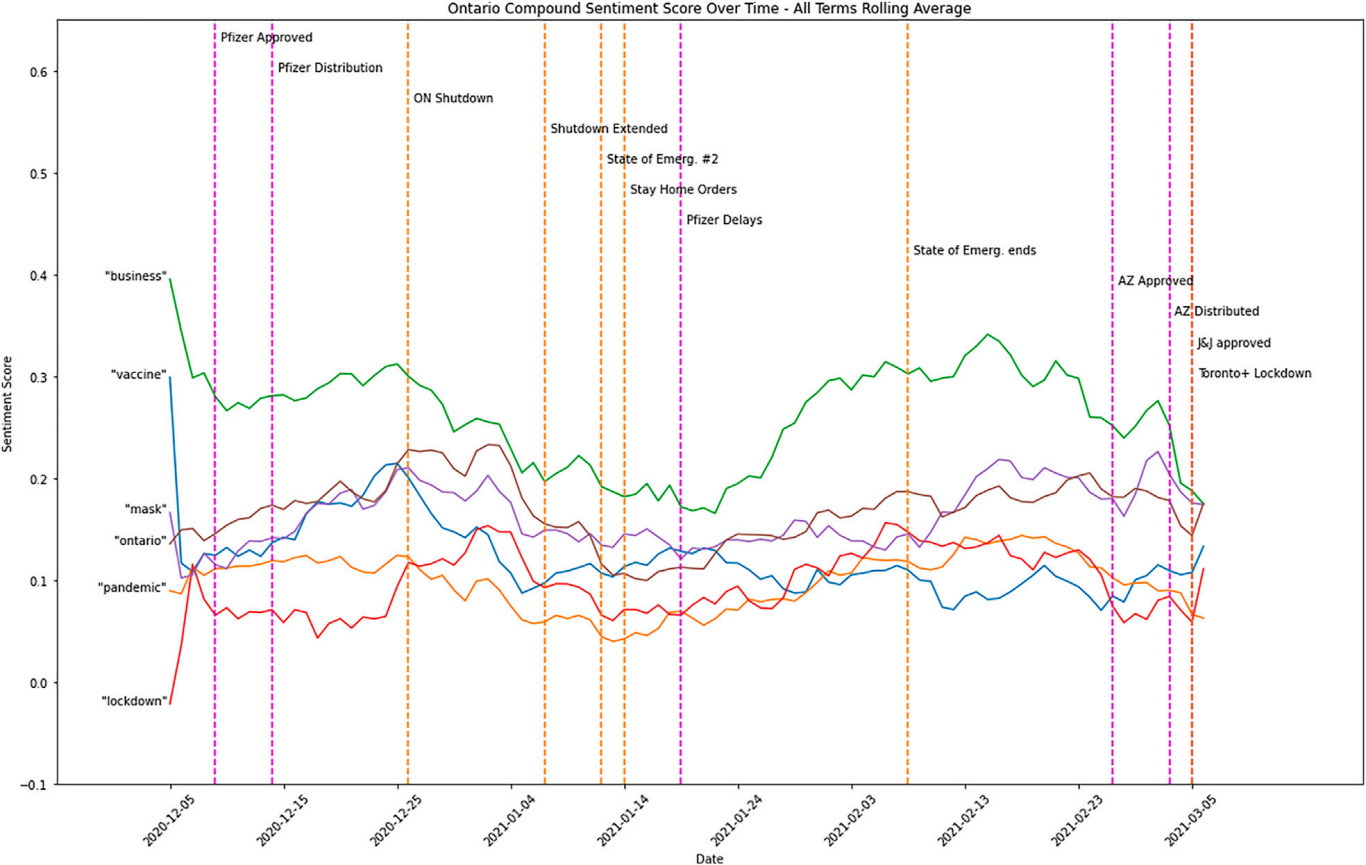
The overall sentiment compund score [Public Attitudes during the Second Lockdown: Sentiment and Topic Analyses using Tweets from Ontario, Canada, Canada, 2020].

The numbers of active COVID-19 cases and deaths between December 5, 2020, and March 6, 2021, in Toronto and Ottawa, Ontario, are shown in Figure 2. Figure 3 demonstrates the sentiment score for each identified topic altogether. Compound sentiment scores for each topic in Ontario are shown in Supplementary Figures S4–S9 in Supplement B, with purple lines representing vaccine events, orange lines indicating policy change events, and red lines demonstrating extended lockdown in Toronto and Peel regions. The light green line shows the mean compound sentiment score for that topic on a particular day, while the dark green line shows the sentiment score smoothed by averaging using an 11-day rolling window. Examples of positive and negative tweets for each topic are shown in Supplementary Tables S2–S7 in Supplement C.

The average sentiment compound score for the topic “lockdown” (in Supplement B Supplementary Figure S4) started neutral but remained slightly positive afterwards. It went up during the Christmas 2020 break and went down after the second provincial emergency was declared.

The average sentiment compound score for the topic “mask” (in Supplement B Supplementary Figure S5) has remained slightly positive during the study period. Similar to the previous topic, the score went up during the Christmas 2020 break and also spiked after the AstraZeneca vaccine was approved in Canada, but it went down shortly afterwards.

The average sentiment compound score for the topic “Ontario” (in Supplement B Supplementary Figure S6) has overall remained slightly positive during the study period, while it consistently declined and plateaued until the second state of emergency ended.

The average sentiment compound score for the topic “pandemic” (in Supplement B Supplementary Figure S7) has been almost neutral over time. However, the score especially decreased after the Pfizer vaccines were delayed and toward the end of the study period.

The average sentiment compound score for the topic “vaccine” (in Supplement B Supplementary Figure S8) began relatively more positive but went down quickly afterwards. It went up shortly before the Christmas 2020 break but declined and plateaued after that.

Like the “vaccine” topic, the average sentiment compound score for the topic “business” (in Supplement B Supplementary Figure S9) started positively but stably dropped afterwards until around January 24, 2021, when the COVID-19 case counts were relatively lower (Figure 2). Interestingly, the average sentiment compound score of the topic “business” was slightly more positive than all the other topics. However, businesses should have been negatively impacted by the lockdown.

## Discussion

To our surprise but understandable, there was no obvious correlation between sentiments and cases or key events on average when the pandemic had lasted over 6 months when Ontario, Canada declared its second provincial emergency and lockdown. Sentiments were more related to holidays as positivity was higher from Christmas 2020 to New Year Day, 2021, although the second provincial shutdown kicked in on December 26, 2020. As Table 1 shows, the “Ontario” topic includes the highest number of tweets, followed by the “pandemic” topic. Regardless of vaccine or policy events, the average sentiment compound score during the studied period appeared to be slightly positive across all topics of interest, with daily sentiment compound scores greatly varied between positive and negative emotions (Supplementary Figures S4–S9). Positive sentiments spiked on December 25, 2020 (Christmas). However, when the second province-wide lockdown began on December 26, 2020, the sentiment scores went downward regardless of topics. In other words, during the second provincial lockdown, there were weak correlations between sentiment scores and vaccine or policy events.

Positive sentiments across topics are mainly associated with holidays and support for the recommended public health practices such as wearing masks and vaccinations. In contrast, negative sentiment across topics largely reflected frustrations and blame on incompetent political leadership. It is also interesting to observe that Greg Abbott, Texas Governor in the United States, was negatively discussed on Twitter among Canadian users when he lifted the mask mandate in Texas on March 2, 2021 [29]. He was a major subject for several days for “business” and “mask” topics.

We did not expect the unsupervised LDA approach for topic modelling to show very limited interpretability in our study. In Table 1, we identified just 66,543 out of 710,936 (9.36%) tweets that can be understood and grouped into six meaningful topics related to our research questions. While we anticipated that the unsupervised ML approach would not generate results as meaningful as supervised ML techniques in previous studies [3–6], we did not foresee such restricted interpretability. Therefore, for future studies, we recommend collecting more data and more thorough data preprocessing to achieve higher data quality to train the unsupervised algorithm and avoid “garbage in, garbage out.” Additionally, for future sentiment analysis, it would be better if emojis could be included to further improve the sentiment scores with a caution that emoji usage can be culturally sensitive in a multicultural country like Canada.

Most existing studies used tweets no later than August 2020, when the first wave of the COVID-19 pandemic was over. In addition, most countries had several lockdowns during the first wave [3–6]. However, in contrast to previous studies showing consistently negative sentiments during the first wave [3–6], our tweets showed that Ontarians had a slightly positive sentiment on average during the second wave in Canada. Unlike previous studies [3–6], symptoms, severity, and/or spread of COVID-19 pandemic are no longer major topics in our sample, demonstrating that Twitter users might have accepted and adjusted daily life under the ongoing pandemic. Similar to existing research showing negative emotions toward government responses [30], especially shutdowns during the first wave, negative tweets in our sample also called political leaders “incompetent” or “failure” across all topics during the second wave, but the average sentiment compound score remains slightly positive, although the daily sentiment compound scores vary a lot.

In addition, our study collected and analyzed tweets over 3 months, which is very different from existing research that collected and analyzed data rarely more than a month [3–6]. Although we did not collect all the possible data given our geolocation restrictions, our research has provided local evidence solely from an aspect of Ontario, Canada, because each country and sub-national entities are unique. This is different from current global sentiment studies that combine and compare different countries together [30–32]. Furthermore, our research is one of a few studies [19] that demonstrated a combination of unsupervised topic modelling and qualitative checks, which can generate human-interpretable and meaningful topics or insights from large amounts of data under a time-sensitive nature without an extremely time-consuming process. However, to avoid “garbage in, garbage out” resulting from the unsupervised ML approach, it remains an important issue to properly specify inclusion and exclusion criteria for data collection, such as keywords or geolocation, to achieve optimized data quality by filtering out meaningless data as much as possible without introducing selection bias in the data.

However, our study has several limitations. First of all, due to Twitter’s geolocation query methods, only a subset of tweets has geotags identified. It has been estimated that only about 2.31% of tweets with locations are attached to the tweets, and an even smaller number of those have precise locations [33]. As such, the tweets we were able to identify and use in our analysis represent only a small amount of the actual discourse occurring within the defined temporal and geographical parameters. Additionally, we limited our search to two very specific municipalities—Toronto and Ottawa in Ontario, Canada—because, during the data collection period, we realized that it was difficult to distinguish “Ontario, Canada” from “Ontario, California” given that their common abbreviation is “Ontario, CA” if we did not specify cities. We also did not collect data from other social media platforms, such as Facebook and Reddit, which has limited our generalizability.

Furthermore, our manual quality validation identified that although small, there is a chance that VADER can misclassify sentiments. For instance, a tweet with sarcasm was regarded as positive but in fact, it should have been considered negative. On the other hand, a tweet with a “surprised” mood was regarded as negative because the word “shocking” was repeated many times. Therefore, it is possible that our sentiment analysis is not perfectly accurate as it should have been. Moreover, tweets classified in each topic were not exclusive to other topics. If a user mentioned several topics within a tweet, it would be assigned to multiple topics, such as the case of Greg Abbott that showed up in both “Ontario” and “business” topics. However, we decided to leave them as they were because it would be inappropriate to assign only one topic to tweets with multiple topics as they naturally occurred. This observation actually showed that some topics could be highly correlated. Accordingly, how to properly choose keywords and their synonyms as filters remains an important challenge to be addressed.

In conclusion, our results have shown that Ontarians in Toronto and Ottawa have remained a slightly positive sentiment during the second wave of the COVID-19 pandemic regardless of topics. We also identified six topics that emerged from data over time, and these topics have been highly correlated with the ongoing pandemic, although the average positive sentiment could be driven by the Christmas-New Year holiday break amid the second wave rather than by the public health interventions. Compared with prior studies conducted during the first wave [19], our study has shown different narratives from public discourse during the second wave. That is, people have shifted their focus on COVID-19 related symptoms, transmissions, risk factors, and origin of the virus to how the pandemic has influenced their daily life without specifically mentioning COVID-19. Our research also demonstrated that a mixed approach of unsupervised topic modelling and manual validation could generate timely evidence when experienced experts get involved. However, data quality and limited utility from unsupervised LDA modelling remain a critical issue for future research, and the possibility of misclassification is acknowledged. Therefore, our results show that it is feasible to use social media data to practice social listening as recommended by the World Health Organization (WHO) to understand narratives from the general public [9] to make informed decisions.

## Abbreviations

COVID-19, Coronavirus disease; LDA, Latent Dirichlet allocation; ML, Machine Learning; VADER, Valence Aware Dictionary and sEntiment Reasoner.
