# Supplementary material for: Public Attitudes During the Second Lockdown: Sentiment and Topic Analyses Using Tweets From Ontario, Canada
Source: Int J Public Health. 2022 Feb 21;67:1604658. doi: 10.3389/ijph.2022.1604658 (PMC8900133; doi:10.3389/ijph.2022.1604658)
Supplement: Supplementary file 1 [file DataSheet3.docx]

# **Supplement C: Tweet Examples**

**Table S3. Examples of positive and negative tweets about topic “lockdown”**

| Positive Tweets | Negative Tweets |
| --- | --- |
| It was tough and it was frustrating but gratefully we mark ourselves safe from this year. Wishing you all Love, Happiness, Health, Safety, Wealth & Peace of Mind. Happy New Year Everyone 🥳 FU 2020 we’re celebrating in lockdown🙃 https://t.co/c1of4YBMDl | You can stay home. We are suffering the worst lockdown than any city in North America, with complete disregard for the lives and livelihoods destroyed. Lockdowns kill more than covid, cancer, suicide, poverty, depression, overdoses, obesity, all of those deaths count too. |
| To all friends &amp; followers facing a holiday season in lockdown… Please stay safe & enjoy your holidays as best you can. We all want to be here together in 2021 ! 🤗💫💗💖🤞🏻 | The POS in over 7%!!? WTF!!! Lockdown on...Boxing Day... Delay vaccines that only last a month. For what?? So you guys can have xmas vacation?? You think soldiers fighting in wars do this shit? Ford is new age stupid. FU #VoteFordOut2022 |
| Wishing you all a very Merry Christmas! Stay safe, be smart, and enjoy today with your family if you can. This time of year can be tough for a lot of people. My DM’s are opened if you need company or wanna chat! Love you guys! 🥰 | Covid is killing people. People that would have died of another complication if not from covid. Think about that in 3 years from now, when Canada is destroyed beyond recognition from the worst depression ever known. The lockdowns saved no one. https://t.co/dQz0staiNd |

**Table S4. Examples of positive and negative tweets about topic “mask”**

| Positive Tweets | Negative Tweets |
| --- | --- |
| That is sad! Everyone has to wear masks where I live in Canada. Stay strong my friend. Hugs 💜💙💜💙💜💙💜💙💜💙💜💙💜💙💜💙💜💙💙💜😙 https://t.co/STuGjEMYB5 | These stupid masks make throat very dry and irritated which is called "sore throat" & it's part of https://t.co/d4hxNq3qs9 made flu to kill 3.5 million people around the world. Leaders have killed approx. 1.7 million.Oh Lord! Forgive these leaders who are lying & killing seniors |
| I love this mask! ❤🧡💛💚💙💜 https://t.co/KBTSs9bazA | WHY would Gov Abbott open business and lift masks when Texas is the 3RD WORST STATE WITH 44,625 PEOPLE HE'S KILLED. Abbott knows it will cause riots, people will get hurt, possible deaths when businesses refuse entry.Then Abbott will put the blame on the people NO WAY ABBOTT |
| Absolutely love them!! Comfortable, extremely soft and fit nicely on the face. Love the adjustable feature! Beautiful masks, thank you | The @premierleague needs to STOP with these fans in the stands. Too close/no masks. Shocking. We’re still in the pandemic!! I don’t want football to be forced to stop this nonsense 😩😩😩 ugh |

**Table S5. Examples of positive and negative tweets about topic “ontario”**

| Positive Tweets | Negative Tweets |
| --- | --- |
| HAPPY VALENTINE'S DAY 💗 💕 💓 💖 💛 ❤ "HEUREUSE SAINT-VALENTIN 💗 💕 💓 💖 💛 ❤ \n 🍾 🍾 🍺 🍻 🍷 🍷 🥂 🎊 🎉!" @ Toronto, Ontario https://t.co/OaXIwwWGbN | 😡 WHAT A TOTAL LOAD OF CRAP..🐾😢💔 😐ONTARIO, YOU SUCK .. YOU DON'T KILL THEM BUT YOU LEAVE A TRAIL OF HEARTBROKEN FOLKS IN YOUR EVIL WAKE DUE TO THESE DUMBASS, NO-BASIS DECISIONS.🐾😢💔 |
| Family Day & Show Day all at once. ☺️☺️☺️ Can’t wait to see you this afternoon, Yellow and Blue Crew. We have a few tickets left! Link in bio. Happy Family Day 💙💛💙 💙💛💙💛💙 @ Toronto, Ontario https://t.co/vYpzarKxTW | Puppet Trudeau and Ford will not make Canada a socialists Country. Trying to follow AmericanDemocrats and China's footsteps. If they're not happy they should move to China. I'm not the virus doesn't exist but very much exaggerated. No more heart attacks, flu, cancer etc., WAKE-UP |
| I LOVE OUR PEOPLE😂😂😂😂😂😂😂😂😂😂😂😂😂😂 , Are we the funniest people on the planet? @ Toronto, Ontario https://t.co/3414PicjyA | This practice of killing horses for human consumption is immoral and should be illegal. Horses are intelligent companion animals.Ontario has been killing horses for many years. This gruesome practice of killing horses and/or transporting them to be killed must stop immediately. https://t.co/1KAdx4iHO3 |

**Table S5. Examples of positive and negative tweets about topic “pandemic”**

| Positive Tweets | Negative Tweets |
| --- | --- |
| For the last decade we’ve used our sign to make the #community smile and send positive vibes! Throughout the pandemic, we knew it was more important than ever to share kind words and motivate people with messages of love, support ♥️🐶♥️🐾 #ThankYou for featuring us.❣️❣️ https://t.co/NSwqUgsThi | Like imagine if they said no 😭😭😭 during a pandemic 😭😭😭😭😭😭 because i went one semester over the limit 😭😭😭😭😭😭😭😭😭😭 Pray for me !! |
| Shoutout to our good friends at for all that they’ve done this year to support the people and #Businesses of #Toronto! 💙🥳🧡🥰 They helped make and distribute hand sanitizer at the onset of the the pandemic to ensure that everyone who needed some had access!❤️ https://t.co/9YVOaYYUnn | I’m sure I’m not the only one in this situation, but I hate it so much. I hate how I’m handling it. Fuck this pandemic. Fuck my weakness. Fuck it all to hell. |
| Its little things like these that make you a STAR!!! Not just your movies but what you do outside, in the real world, that's the most powerful impact on our lives!! Always love and respect for you sir. Hope you and your loved ones are safe in the pandemic!! #therock #DwantaClaus https://t.co/us5fmy5VWm | Trying to deal with COVID anxiety, and what do I get today? TMJ dysfunction 😭😭😭😭 I hate not doing joint mobilisation, but..but... 😭😭😭😭😭 |

**Table S7: Examples of positive and negative tweets about topic “vaccine”**

| Positive Tweets | Negative Tweets |
| --- | --- |
| Many of my friends and my goddaughter got their vaccines today. Our healthcare workers are heroes. Happy beyond happy for each and every one of you. #HealthcareHeroes 💕💕💕💕 | The POS in over 7%!!? WTF!!! Lockdown on...Boxing Day... Delay vaccines that only last a month. For what?? So you guys can have xmas vacation?? You think soldiers fighting in wars do this shit?\n\nFord is new age stupid. FU\n\n#VoteFordOut2022 |
| The greatest lesson of 2020 is that we need each other—-we revel in each other’s support and rise through lifting each other in unity, we are stronger through collective prosperity and universal love. A united humanity is our greatest vaccine.... #unity #hera #love #Africa https://t.co/6BFrduemEm | Leader? No not really, anyone that stoops to the bottom feeding levels that he does by lying to Cdn's and attempting to scare them with a fake vaccine scare is no a leader, he is a bad human. #cdnpoli |
| To any educator out there: congratulations on making it to the most TGIF in the history of TGIFs!!! You are all my heroes. Your efforts do not go unnoticed and should be rewarded with early vaccinations. Happy holidays! https://t.co/MRleMMtZuO | it is impossible to understand what is happening. people die in isolation while their traumatized families struggle for morsels of information and vaccines sit in refrigerators. This is incompetence at its most devastating. #ontario #onpoli https://t.co/vordYwDHVB |

**Table S8. Examples of positive and negative tweets about topic “business”**

| Positive Tweets | Negative Tweets |
| --- | --- |
| These keychains are perfect gifts for anyone that loves hearts, they can be made in (almost) any colour, with or without the boarder/frill. ❤️ 💜 💙 💚 💛 🧡 🖤 🤍\nDM me for commissions or questions :)\n#smallbusiness #crochet #customgifts #yarnspirations https://t.co/ufOOiXgowS | In the coming months we will play catch up but the harm being done today to the vulnerable, to businesses, to the mental health of young people is indelible. Each day lost to Covid adds more damage, more suffering, more economic hardship, more death. That’s on Trudeau forever. https://t.co/1qCwZ9UgP6 |
| Its great to spend time entertaining ourselves with movies, sports, etc. But spending time on a #journey to become our better #self... that's the #best!\n#nature #mentor #success #journey #inspiration #motivation #amazing #love #outdoors #out #business #SelfImprovement #mentorship https://t.co/BWt7KR2wWM | DUMBASS GOV GREG ABBOTT DOESN'T CARE LESS ABOUT TEXANS. HIS OPENING ALL BUSINESSES AND NO MASKS IS RECKLESS.\n\nCOVID-19 AND THE VARIANTS ARE GOING TO SKYROCKET. WHO WILL DUMBASS ABBOTT TRY TO BLAME IT ON ???\n\nGOV ABBOTT WILL BE SOLELY RESPONSIBLE FOR EVERY SINGLE DEATH !!!! |
| Wishing everyone a Happy and Safe New Year! Thank You all for supporting my small business and Dream!!! Thanks to All my Customers and Fans, Super sexy Models and Talented Photographers and most of all, Thank you to my small work staff. Love you all!!! ❤️🥂 2021 #RawStudioDesigns https://t.co/rNieZmczFn | WHY would Gov Abbott open business and lift masks when Texas is the 3RD WORST STATE WITH 44,625 PEOPLE HE'S KILLED. Abbott knows it will cause riots, people will get hurt, possible deaths when businesses refuse entry.Then Abbott will put the blame on the people NO WAY ABBOTT |
