# Supplementary material for: Public Attitudes During the Second Lockdown: Sentiment and Topic Analyses Using Tweets From Ontario, Canada
Source: Int J Public Health. 2022 Feb 21;67:1604658. doi: 10.3389/ijph.2022.1604658 (PMC8900133; doi:10.3389/ijph.2022.1604658)

# **Supplement B: Sentiment score for each topic**

Figure S4: Sentiment Compound score for the topic “lockdown”


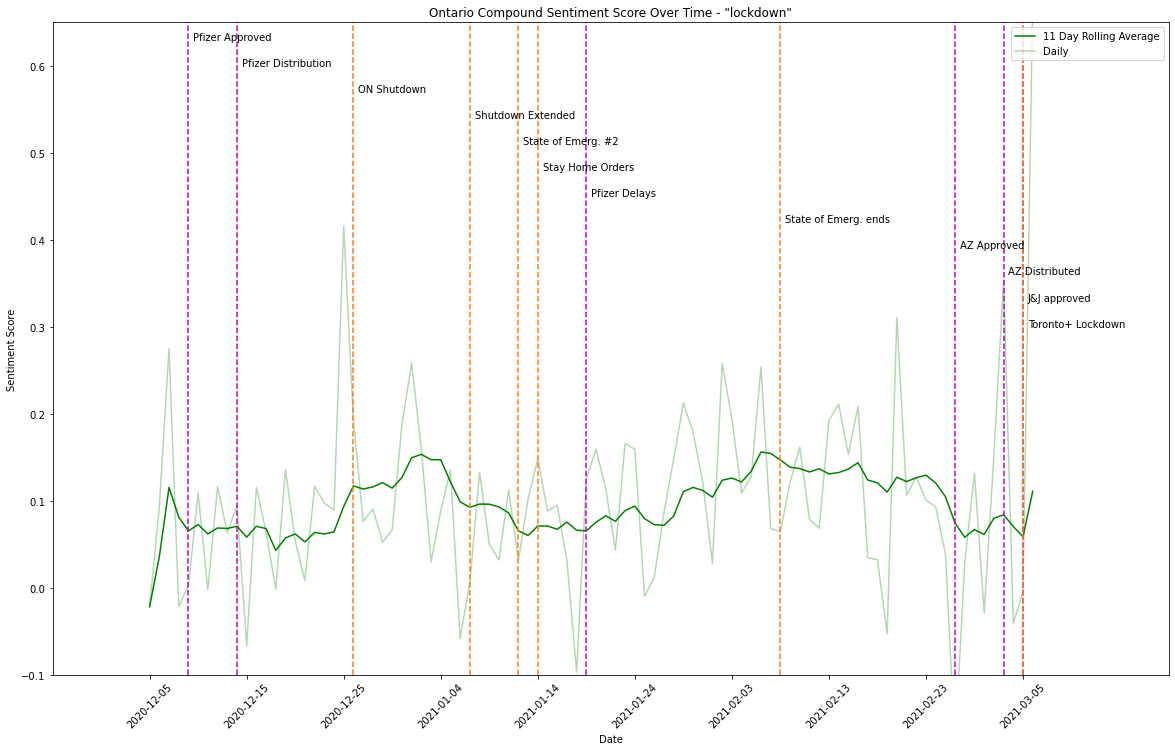


Figure S5: Sentiment Compound score for the topic “mask”


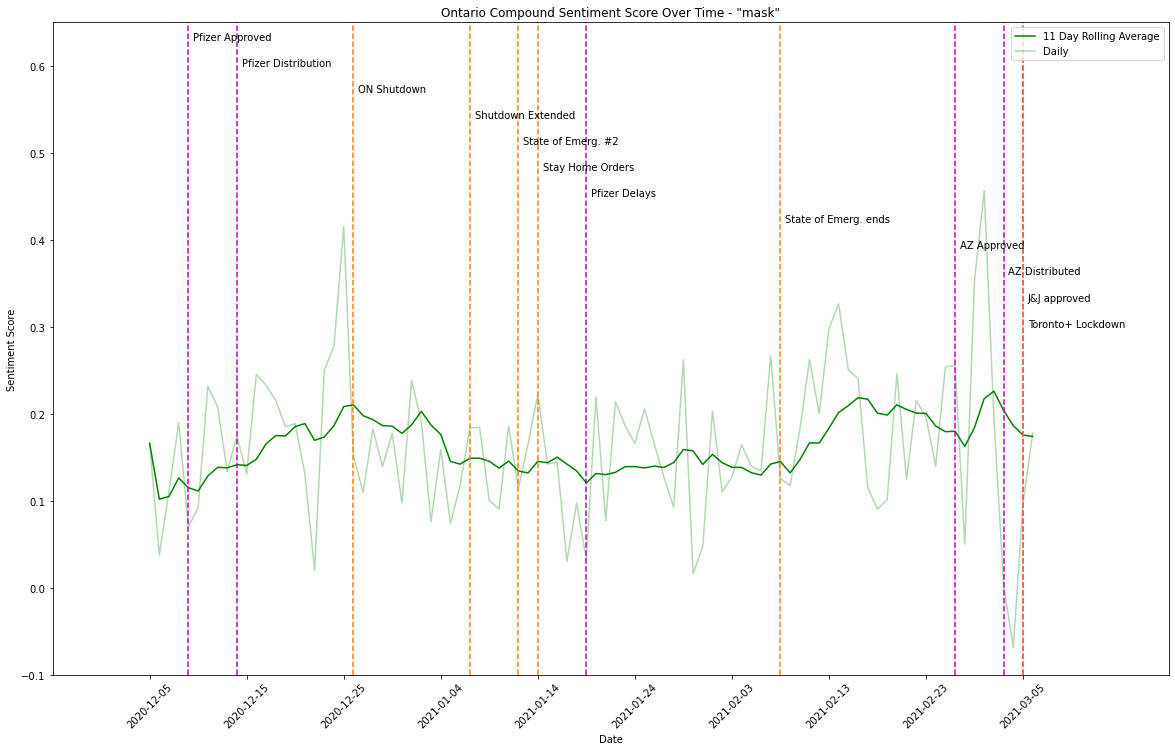


Figure S6: Sentiment Compound score for the topic “ontario”


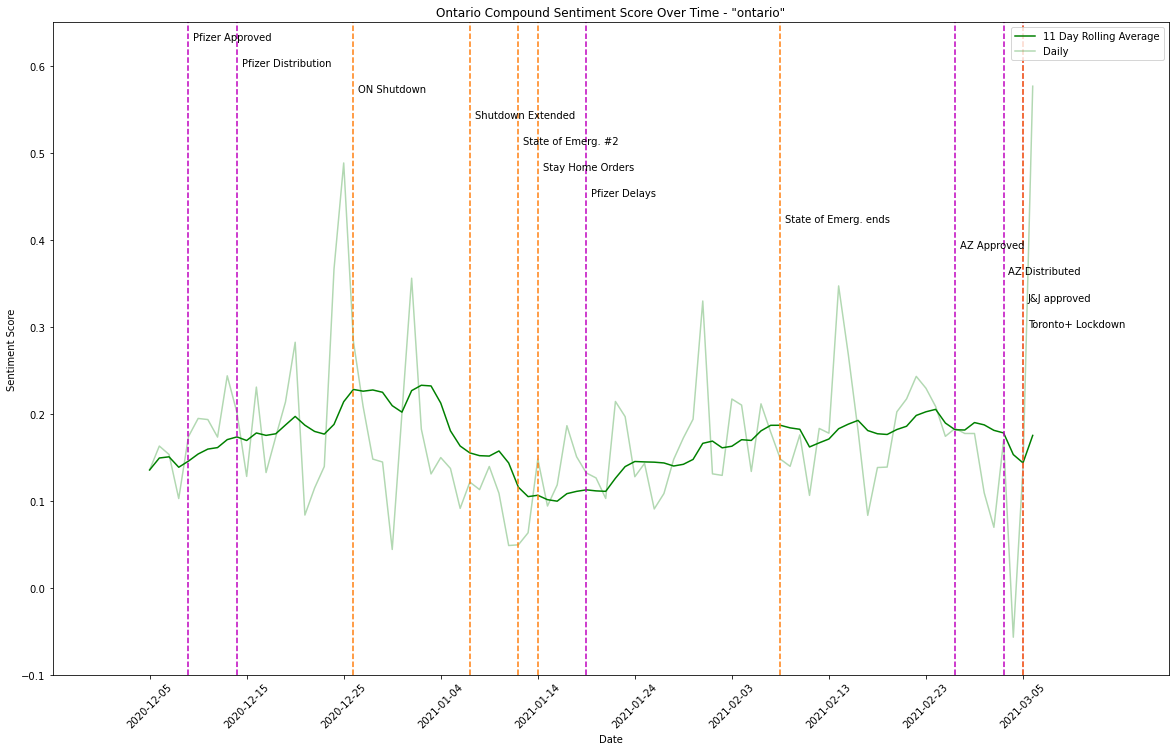


Figure S7: Sentiment Compound score for the topic “pandemic”


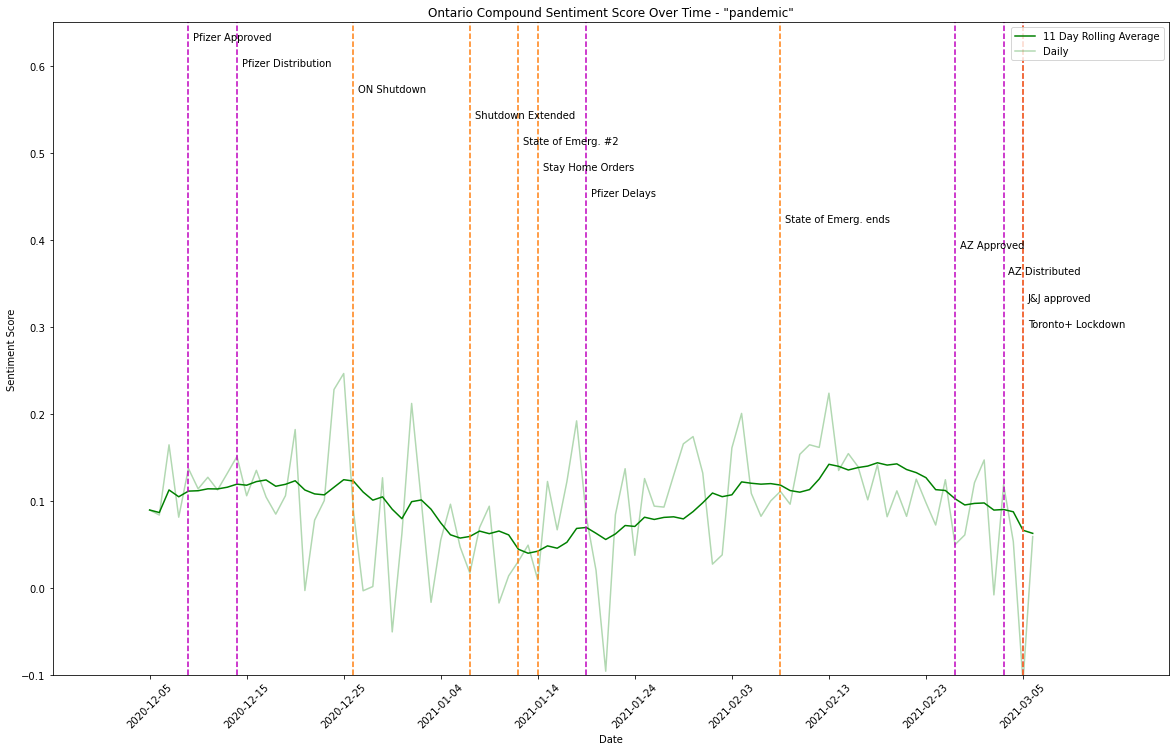


Figure S8: Sentiment Compound score for the topic “vaccine”


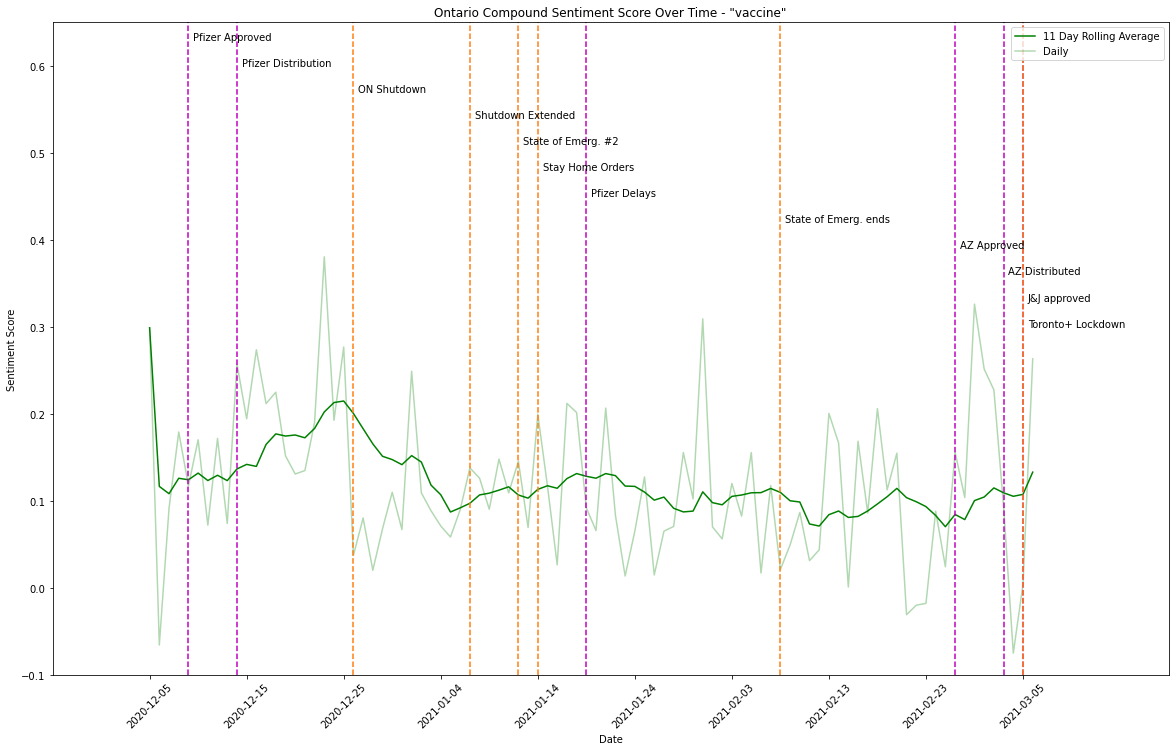


Figure S9: Sentiment Compound score for the topic “business”


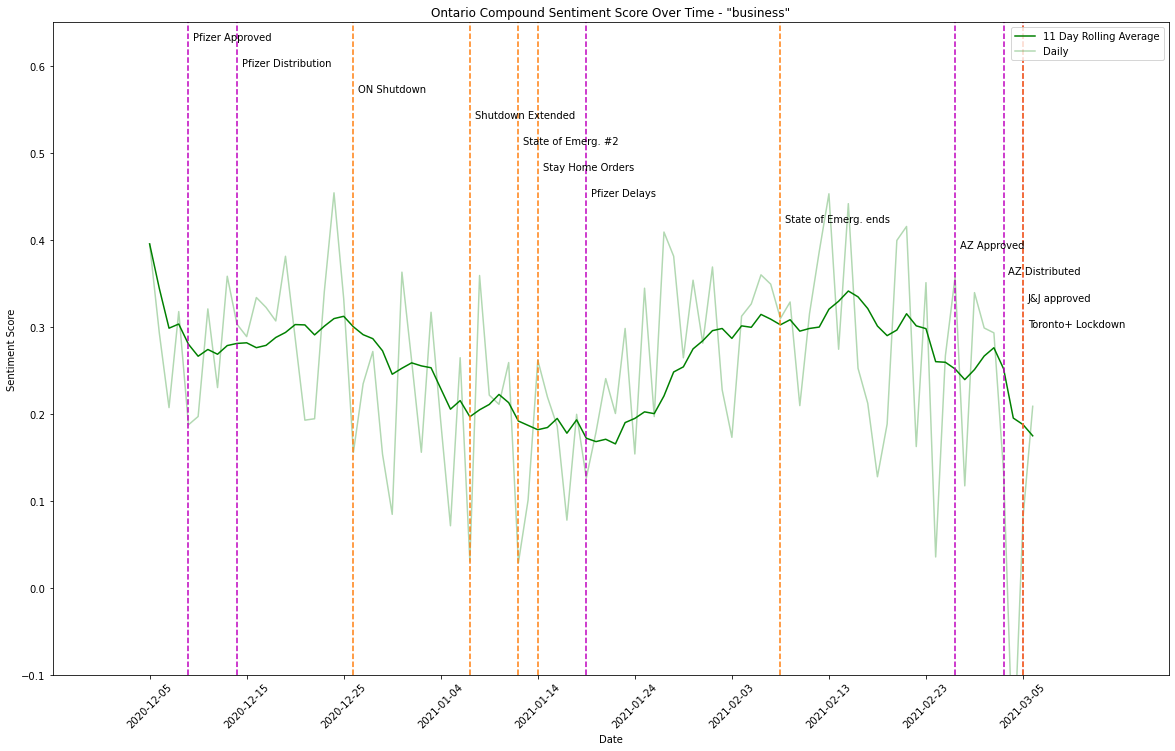

Supplement: Supplementary file 2 [file DataSheet2.docx]
