# Supplementary material for: Public Attitudes During the Second Lockdown: Sentiment and Topic Analyses Using Tweets From Ontario, Canada
Source: Int J Public Health. 2022 Feb 21;67:1604658. doi: 10.3389/ijph.2022.1604658 (PMC8900133; doi:10.3389/ijph.2022.1604658)
Supplement: Supplementary file 3 [file DataSheet1.docx]

# **Supplement A: Inter-rater agreement**

The inter-rater agreement percentages and validation results were shown in Table S1 and 2, respectively. Overall, the average inter-rater agreement percentages and accuracies for topics amd sentiments are reasonable. As Table 2 and 3 show, the inter-rater agreement percentages and accuracies for topics are, on average, higher than those for sentiments. However, certain topics have lower-than-average topic accuracy. For example, “mask” has the lowest topic accuracy than all other topics. During the manual validation, we found that although some tweets have the keyword "mask,” it’s used as a verb to critize someone is covering something up. Additionally, some users mentioned that they wore anything else but masks in tweets, but the LDA model still grouped these tweets together, resulting a low topic accuracy. For topics with lower-than-average sentiment accuracy, it’s generally resulted from tweets with ironic or sarcastic expressions, espcially when Twitter users critized political leaders’ performances.

**Table S1 Inter-rater agreements for topics and sentiments**

| Topic | Primary rater | Secondary rater | Inter-rater agreement for Topic (%) | Inter-rater agreement for Sentiment (%) |
| --- | --- | --- | --- | --- |
| business | SFT | HC | 100 | 93 |
| lockdown | SFT | ZB | 100 | 80 |
| mask | SFT | HC | 100 | 77 |
| ontario | SFT | ZB | 100 | 83 |
| pandemic | SFT | HC | 100 | 90 |
| vaccine | SFT | ZB | 100 | 67 |
| Average | | | 100 | 82 |

**Table S2 Topic and Sentiment Manual Validation**

| Topic | Topic Accuracy (%) | Sentiment Accuracy (%) |
| --- | --- | --- |
| business | 78 | 80 |
| lockdown | 81 | 73 |
| mask | 56 | 93 |
| ontario | 92 | 63 |
| pandemic | 97 | 60 |
| vaccine | 99 | 70 |
| Average | 84 | 73 |
